# Success Rate of Microimplants in a University Orthodontic Clinic

**DOI:** 10.5402/2011/982671

**Published:** 2011-04-26

**Authors:** P. Sharma, A. Valiathan, A. Sivakumar

**Affiliations:** ^1^Department of Orthodontics and Dentofacial Orthopaedics, Manipal College of Dental Sciences, Manipal University, Manipal 576104, KRN, India; ^2^Department of Orthodontics, Al-Fateh University of Medical Sciences, P.O. Box 13040, Tripoli, Libya

## Abstract

*Introduction*. The purpose of this study was to examine the success rate and find factors affecting the clinical success of microimplants used as orthodontic anchorage. *Methods*. Seventy-three consecutive patients (25 male, 48 female; mean age, 22.45 years) with a total of 139 screw implants of 2 types were examined. Success rate was determined according to 18 clinical variables. *Results*. The overall success rate was 87.8%. The clinical variables of microimplant factors (type), patient factors (sex, skeletal and dental relationships, overbite, jaw involved, side involved and site involved), and treatment factors (type of insertion, time of loading, purpose of microimplant insertion, mode of loading, type of anchorage used, direction of forces applied) did not show any statistical difference in success rates. Mandibular angle, vertical position of implant placement, oral hygiene status, and inflammation showed significant difference in success rates. *Conclusions*. Proper case selection and following the recommended protocol are extremely essential to minimise failures.

## 1. Introduction

Anchorage has always been a matter of great concern in orthodontics. Extraoral and intraoral anchorage methods have stood the test of time in providing the best possible treatment outcomes except for some cardinal limitations like compliance factor, slippage, and so forth. Osseointegrated implants have also long been used for a variety of purposes [1] including anchorage. The introduction of skeletal anchorage system (SAS) by Sugawara [2], microscrews by Kanomi [3], and the micro-implant anchorage (MIA) by Park [4] and Park et al. [5] in the orthodontic treatment care has negated the limitation faced by other anchorage devices. 

Clinical applications of microimplants as direct or indirect anchorage methods include correction of deep bite, closure of extraction spaces, correction of canted occlusal plane, alignment of dental midlines, extrusion of impacted canines, extrusion and uprighting of impacted molars, molar intrusion, distalization of either maxillary molars or mandibular teeth, en-masse retraction of anterior teeth, molar mesialization, maxillary third molar alignment, intermaxillary anchorage to correct sagittal discrepancies and correction of vertical skeletal discrepancies. 

The term “Temporary Anchorage Devices” (TADs) has become quite popular today and it connotes the nature of MI as a nonosseointegrated device made of titanium alloy or stainless steel meant only for anchorage purpose during active tooth movement. Thus, the common concern revolves around their efficiency, that is, the success of micro-implants is dependent on their holding in the bone as stationary anchors. Hence, an evaluation of the success rate in a systematic protocol is absolutely essential. Recently, some investigations have evaluated the failure or success rates of micro-implants and risk factors associated with their use as TADs for orthodontic purposes [6–13]. According to these studies, the success rates have varied long between 75.2% and 90.7%. The aim of this retrospective study was to find factors related to the clinical success of micro-implants in Asian patients.

## 2. Material and Methods

Case history and treatment detail records for cases treated using micro-implants from 2007 to 2009 at our university orthodontic clinic; and self-made questionnaire containing the relevant study variables were employed for the study. The questionnaire had been filled by the treating faculty members and post-graduate students who had formal training in microimplant insertion. The database was generated with the details recorded using Microsoft Office Excel 2007. In total, 139 microimplants were placed in 73 patients, including 25 males and 48 females. The ages ranged from 13 to 50 years, with a mean of 22.45 years. Before implantation, all these patients were informed of the advantages and disadvantages associated with the use MI as part of orthodontic treatment strategy. 

The microimplants used in the clinical set up include Indian Implants (S.K. Surgicals, Pune) and Absoanchors (Dentos, Daugu, South Korea). They are composed of Titanium-6Aluminum-4Vanadium ELI Alloy material. The screw dimensions considered were 1.3 mm diameter and 8 mm length. Recommended guidelines were followed for the insertion procedure. Topical anaesthesia was applied before implant placement. The screw implants were placed at 30° to 40° angles to the long axes of the teeth in the maxillary arch and at 10° to 20° angles in the mandibular posterior area according to the recommendations of the clinicians who developed them [13]. No analgesics or antibiotics were prescribed after microimplant placement. Postoperative discomfort was minimal for all patients. The patients were instructed to gently massage the implant area and to keep it clean. A chlorhexidine rinse was prescribed for 7 to 14 days. To check the effect of oral hygiene on success, the amount of food debris and plaque accumulation on the tooth surfaces was assessed; the sample was divided into 3 groups: good, fair, or poor. Inflammation around the screw implant was checked in the following categories: yes or no. Redness or swelling around the neck of the screws was a sign of inflammation.

Orthodontic force was applied either immediately or after initial wound healing depending on the clinical situation and operator's skill. Elastomeric modules (Libral Traders, New Delhi, India), power chain or closed-type NiTi coil springs (Libral Traders, New Delhi, India), stainless steel closed coil (H.P. Industries, India) or ligature wire (Libral Traders, New Delhi, India) for activation of loop were used as the source of orthodontic traction force. Forces were applied either directly to the microimplants (direct anchorage) or indirectly (indirect anchorage) to achieve better torque and directional control and to prevent gingival impingement. These forces were used for the following orthodontic purposes: (1) en-masse retraction of the 6 anterior teeth in arches, (2) distalization of the molar teeth (3) protraction of the molar teeth (4) intrusion of the maxillary and mandibular incisors and molars, and (5) en-masse distalization of the dental arches as per the requirement of the case.

The criteria of microimplant success were: (1) no inflammation of the soft tissues surrounding the microimplant, (2) no clinically detectable mobility, and (3) anchorage function sustained until the end of the purpose for which the implant was used.

Failure was defined as spontaneous loss, severe clinical mobility of the microimplant requiring replacement, or infected, painful, pathologic changes in the surrounding soft tissues. 

Clinical variables influencing the success rates of the microimplants were categorized as patient-related, implant-related, and treatment-related and assessed accordingly.

*Patient-related factors* included sex, skeletal and dental relationships (Class I, Class II, or Class III), mandibular angle (high, average or low) overbite (increased, decreased, or normal), oral hygiene status (good, fair or poor), jaw involved (maxilla or mandible), side involved (right or left), and site involved (anterior to premolar area, posterior to premolar area, retromolar area or palatal area). 

*Implant-related factors* included implant type (Indian implant or Abso Anchor).

*Treatment-related factors* included type of insertion (self drilling or self tapping), vertical position of insertion (attached or moveable gingiva), time of loading, purpose of microimplant insertion, mode of loading, type of anchorage used, direction of forces applied (horizontal, vertical or both), and any inflammation if present.

## 3. Statistics

Descriptive statistics were initially performed for each factor. Fisher's exact test was used when there was an expected value <5 in any cell of the cross-tables while chi-square test was conducted for others. The data were statistically analyzed with SPSS software (version 11.5, SPSS, Chicago, Ill). The statistical significance was set at *P* < .05 for all tests.

## 4. Results

The overall success rate was 87.8% for all microimplants (122 of 139). The microimplant survival time was (8.96 ± 4.8) months. The data regarding overall and detailed success rates of microimplants patient-, implant-, and treatment-related factors are shown in Tables 1 and 2. 

Of the patient related factors, there were no significant differences according to sex, jaw, side and site of placement, skeletal or dental relationship, and overbite. However, patients with a poor oral hygiene and high mandibular angle showed significantly less success than those with a good oral hygiene and low mandibular angle. Male patients showed a higher success rate than female patients though it was not significant statistically. Patients with a Skeletal Class I relation showed higher success than those with Skeletal Class II and Class III relations though there was no statistical significance.

For implant-related factors, there were no significant differences in the success rate of the two types of implants studied: Type A (Indian Implant) Type B (Absoanchor).

Among treatment related factors, there was no significant correlation in success rate according to the type of microimplant insertion (self drilling or self tapping), time of loading, purpose of microimplant placement, mode of loading, direction of forces and type of anchorage used. The implants placed in the attached gingiva had significantly higher success than those placed in the moveable gingiva. Also, microimplants with inflammation showed significantly less success.

## 5. Discussion

Since microimplants are a relatively new innovation, little is known about factors that affect the rates of success of screw implants. Therefore, in this study, we tried to include as many factors as possible. Patient-related, implant-related, and treatment-related factors were evaluated. Among them, significant differences were found among patient related and treatment related factors.

The success rate of microimplants in the present study was 87.8%. The success rate for microimplants in previous studies varied between 83.9% and 93.3% [12–14]. These rates might be explained by the various types of microimplants, different surgical techniques, and varying management protocols. Therefore, a direct comparison of success rates might not be possible. Also, the success rate in this study was comparable with the rates in other studies of Asian patients (75.20%–91.60%) [6–11]. 

Although male patients showed higher success than female patients but this was not significant statistically. This finding was similar to the previous studies [6–11]. 

 Inflammation can damage the bone surrounding the neck of microimplants. With progressive damage of the cortical bone, screw implants can be endangered [6]. To ensure success, it is important to prevent inflammation around the screw implants. In this study, both oral hygiene and local inflammation significantly affected the success rate. In the study by Park et al. [11], oral hygiene did not affect success but local inflammation around the microimplants did. Local inflammation can be exaggerated not only by oral hygiene but also by weak nonkeratinized soft tissue around the neck of the screw implant. A recent study showed that nonkeratinized mucosa was a risk factor for miniscrews [12].

This study showed no difference in success rates of microimplants for maxillary or mandibular placement in contrast to the results of Chen et al. [8] and Park et al. [11], who found lower success rates for microimplants in the mandibles of Asian patients. Cortical bone thickness of the mandible that might cause bone overheating while drilling and the short zone of attached gingiva have been suggested as causes of these significantly lower success rates of microimplants in the mandible. Our results were similar to previous studies by Wiechmannn et al. [10] and Antoszweska et al. [15], that were not necessarily correlated with anatomic factors, such as cortical bone thickness.

Placement of microimplants in the left quadrant of the dental arch, although not significant in our evaluation, seemed to have a higher success rate than on the right side as also reported previously [11]. A possible explanation for this might be better oral hygiene on the left side of the oral cavity that is usually observed in right-handed patients, who are the majority [16].

Furthermore, no association was found between success rate and site of microimplant placement. This could be due to the lower sample size of the study. In previous studies, it was found that microimplants on the right side of the mandible between the first and second molars had significantly lower success rates: Kuroda et al. [7] and Antoszweska et al. [15]. Chen et al. [8] found the lowest success rates on either side of the mandible between the first and second premolars.

There was no statistical significant difference in success rate according to the skeletal and dental relationships. This was in accordance with the previous studies.

Our study observed lower success rates in patients with increased mandibular angles similar to the previous studies by Miyawaki et al. [13] and Moon et al. [6]. The lower success rates of microimplants in patients with open bites or increased mandibular angles might be because these patients usually have thin cortical bones, the volume of which significantly contributes to the mechanical retention of nonosseointegrated microimplants.

In contrast to the study by Antoszweska et al. [15], where open bite was found to be a negative factor significantly influencing the stability of the microimplants, in our study, no statistically significant difference was found between overbite and success rate.

 The study showed no significant difference in success rate according to the type of microimplant and type of insertion (self-drilling or self-tapping). The main difference between self-tapping screws and self-drilling, self-tapping screws is that the former requires a predrilled hole of lesser diameter than the thickness of the screw which is placed and tightened either manually or by a power-driven screw driver. In case of self-drilling, self-tapping screws, no predrilled hole is required, because the screw has a built-in drill point and does the drilling in steel and the tapping on its own in a single operation. This shows that by following proper surgical protocol and following the recommended instructions, one can achieve a good success rate.

With regard to the vertical position of microimplants, their placement in the attached gingiva of the maxilla was associated with significantly higher success rates, in contrast to the results of Park et al. [11], who found higher success rates when microimplants were placed in the oral mucosa of maxilla. However, this is in accordance with the previous study by Antoszweska et al. [15].

There was no significant difference in the success rate with respect to the onset of force application. This might indicate that immediate loading of screw implants is possible. An animal experiment proved that there was osseointegration after immediate loading of the screw implants and suggested immediate loading to reduce the treatment time [17]. Recent reports also recommend immediate loading of screw implants [18]. Therefore, screw implants can be loaded immediately after placement without a discernible deterioration of stability.

No significant difference was found between the success rate and the purpose for which the microimplant was placed. This could be due to the lower sample size of the study. However, this depicts the versatility of microimplants.

There was no stastically significant difference found in success rate according to the mode of loading (similar to Chen et al.) [8], type of anchorage (similar to Antoszweska et al.) [15], and direction of forces. However, Antoszweska et al. [15] showed that vertical forces applied to microimplants seem to be responsible for more failures than horizontal forces. 

One drawback of the study is the lower sample size. This puts limitations to the depth and extent of research of clinical variables studied. Since most of the microimplants used in the department were 1.3 mm in diameter and 8 mm in length, the effects of the dimensions of microimplant on the clinical success were not considered. Majority of the sample comprised of young population; therefore, age factor and systemic problems were not considered. However, the effect of the above shortcomings should be elucidated in a future study.

## 6. Conclusions

The overall success rate was 87.8%. This shows that microimplants can be used for orthodontic anchorage predictably and consistently in routine orthodontic practice. Poor oral hygiene, high mandibular angle, placement in moveable gingival, and inflammation were associated with microimplant failure in this study. Thus, proper case selection and following the recommended protocol are extremely essential to minimise failures.

## Figures and Tables

**Table 1:** Demographic information of 73 subjects and 139 micro-implants in this study.

Clinical variable	Number (%)
Gender (Male/Female)	25/48
Age (years) (mean ± standard deviation)	22.45 ± 6
Skeletal Malocclusion (Class I/Class II/ ClassIII)	42/24/7
Dental Malocclusion (Class I/Class II/ ClassIII)	35/29/9
Number of implants per patient (1/2/4)	23/42/8

**Table 2:** Univariate analysis of factors associated with microimplant success.

Clinical variable	Success rate (%)	Success rate (*n*)	Significance (*P* value)
Overall success	87.8	122/139	
Gender			
Male	95.5	42/44	.06
Female	84.2	80/95	
Oral hygiene			
Good	92	69/75	.01
Fair	96	48/50	
Poor	35.7	5/14	
Jaw			
Maxilla	87.6	85/97	.93
Mandible	88	35/42	
Side			
Right	86.3	57/66	.63
Left	89	65/73	
Site			
Ant. to premolars	94.4	17/18	.59
Post. to premolars	86	99/115	
Retromolar area	100	5/5	
Palatal area	100	1/1	
Skeletal Relation			
Class I	92.8	78/84	.07
Class II	80	36/45	
Class III	80	8/10	
Dental Relation			
Class I	89.1	66/74	.86
Class II	86.2	44/51	
Class III	85.7	12/14	
Mandibular angle			
High (>28°)	72.5	29/40	.006
Average (17–28°)	93.2	82/88	
Low (<17°)	81.8	9/11	
Overbite			
Open (<1 mm)	87.5	14/16	.47
Normal (1–4 mm)	90	81/90	
Deep (>4 mm)	81.81	27/33	
Implant type			
A (Indian)	89.5	86/96	.33
B (Absoanchor)	83.7	36/43	
Type of insertion			
Self drilling	83.7	31/37	.39
Self tapping	89.2	91/102	
Vertical position			
Attached gingiva	91	112/123	.001
Moveable gingiva	62.5	10/16	
Time of loading			
Immediate	89.79	88/98	.26
After 1 week	82.92	34/41	
Purpose			
Mol. protraction	94.4	17/18	.25
Mol. distalisation	100	13/13	
Intrusion	92.8	13/14	
Ant retraction	84	79/94	
Mode of loading			
Ni Ti coil	100	12/12	.49
Stain. steel coil	81.8	18/22	
E-chain	87.5	77/88	
Loop mechanics	88.2	15/17	
Force direction			
Horizontal	88.9	105/118	.26
Vertical	86.6	13/15	
Both	66.6	4/6	
Anchorage			
Direct	87.5	98/112	.84
Indirect	88.8	24/27	
Inflammation			
Yes	50	113/121	.01
No	93.3	9/18	

## References

[1] Ravinder V, Sunny J, D’souza M, Ashima V (2003). Osseo integrated implants for maxillary lateral incisors-orthodontic considerations. *Malaysian Dental Journal*.

[2] Sugawara J (1999). Dr. Junji Sugawara on the skeletal anchorage system. Interview by Dr. Larry W. White. *Journal of Clinical Orthodontics*.

[3] Kanomi R (1997). Mini-implant for orthodontic anchorage. *Journal of Clinical Orthodontics*.

[4] Park HS (1999). The skeletal cortical anchorage using titanium microscrew implants. *Korean Journal of Orthodontics*.

[5] Park HS, Bae SM, Kyung HM, Sung JH (2001). Micro-implant anchorage for treatment of skeletal Class I bialveolar protrusion. *Journal of Clinical Orthodontics*.

[6] Moon CH, Lee DG, Lee HS, Im JS, Baek SH (2008). Factors associated with the success rate of orthodontic miniscrews placed in the upper and lower posterior buccal region. *Angle Orthodontist*.

[7] Kuroda S, Sugawara Y, Deguchi T, Kyung HM, Takano-Yamamoto T (2007). Clinical use of miniscrew implants as orthodontic anchorage: success rates and postoperative discomfort. *American Journal of Orthodontics and Dentofacial Orthopedics*.

[8] Chen YJ, Chang HH, Huang CY, Hung HC, Lai EHH, Yao CCJ (2007). A retrospective analysis of the failure rate of three different orthodontic skeletal anchorage systems. *Clinical Oral Implants Research*.

[9] Luzi C, Verna C, Melsen B (2007). A prospective clinical investigation of the failure rate of immediately loaded mini-implants used for orthodontic anchorage. *Progress in Orthodontics*.

[10] Wiechmann D, Meyer U, Büchter A (2007). Success rate of mini- and micro-implants used for orthodontic anchorage: a prospective clinical study. *Clinical Oral Implants Research*.

[11] Park HS, Jeong SH, Kwon OW (2006). Factors affecting the clinical success of screw implants used as orthodontic anchorage. *American Journal of Orthodontics and Dentofacial Orthopedics*.

[12] Cheng SJ, Tseng IY, Lee JJ, Kok SH (2004). A prospective study of the risk factors associated with failure of mini-implants used for orthodontic anchorage. *International Journal of Oral and Maxillofacial Implants*.

[13] Miyawaki S, Koyama I, Inoue M, Mishima K, Sugahara T, Takano-Yamamoto T (2003). Factors associated with the stability of titanium screws placed in the posterior region for orthodontic anchorage. *American Journal of Orthodontics and Dentofacial Orthopedics*.

[14] Park HS (2003). Clinical study on success rate of microscrew implants for orthodontic anchorage. *Korean Journal of Orthodontics*.

[15] Antoszewska J, Papadopoulos MA, Park HS, Ludwig B (2009). Five-year experience with orthodontic miniscrew implants: a retrospective investigation of factors influencing success rates. *American Journal of Orthodontics and Dentofacial Orthopedics*.

[16] Tezel A, Orbak R, Çanakçi V (2001). The effect of right or left-handedness on oral hygiene. *International Journal of Neuroscience*.

[17] Melsen B, Costa A (2000). Immediate loading of implants used for orthodontic anchorage. *Clinical Orthodontics and Research*.

[18] Park HS (2001). *The Use of Micro-Implant as Orthodontic Anchorage*.

